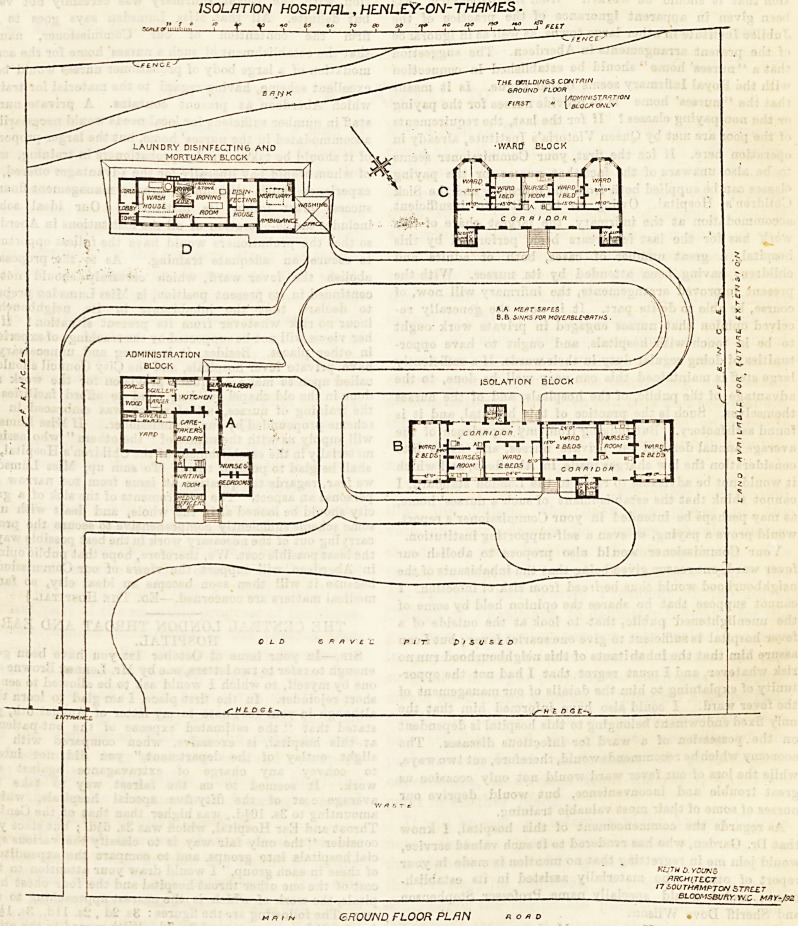# The "W. H. Smith" Isolation Hospital, Henley-On-Thames

**Published:** 1892-10-22

**Authors:** 


### HOSPITAL CONSTRUCTION.

### THE "W. H. SMITH" ISOLATION HOSPITAL, HENLEY-ON-THAMES.

The provision of an isolation hospital for the Rural Sanitary Authority of Henley-on-Thames is due to the large-minded generosity of the late Right Honourable W. H. Smith and of Mr. H. D. Mackenzie, of Fawley Court. Mr. W. H. Smith provided the buildings, with furniture and equipments complete, and Mr. Mackenzie gave the site and the trees and shrubs and the labour connected with laying out the grounds. The site is some acres in extent, only about one-half of which will at present be utilised for hospital purposes, the remainder being reserved for future extension. It is situate about a mile out of the town of Henley, on that part of the Oxford Road known as the "Fair Mile," and is well screened from the road by a lofty row of trees.

The hospital comprises four detached blocks. A. The administration; B. Ward block for ordinary patients; C. Block for paying patients; and D. Laundry, mortuary, &c. The administrative block (A) is two storeys in height. On the ground floor is a small room for the medical officer, entered from the porch, a waiting-room, which will serve also as sitting-room for nurses, two nurses bed-rooms, bed-room for caretaker, kitchen, scullery, larder, and fuel stores. In one angle of the kitchen is a porch, fitted with a revolving serving hatch for the service of meals, &c., to the ward blocks. On the upper floor are four bed-rooms for nurses, a bed-room for servants, and a bath-room.

Block B is a modification of the Local Government Board isolation block, and contains two sets of wards, entered from opposite sides. In each set are two wards for two beds each, with a nurse's room between the two wards. Space for a wheeled bath and a meat safe is arranged in the verandah. To each set of wards a water-closet and slop sink are provided. The wards are lined up to a height of five feet with tinted glazed bricks, above which the walls are distempered. The floors are laid with yellow deal boards in narrow widths, secret nailed. The grates in the walls are Boyd's "Hygiastic," provided with fresh air ducts from outside. The walls of the corridors and the w.c.'s are lined throughout with glazed bricks.

Block C contains a nurse's room and four wards. The two end wards are made somewhat larger than the others in order to accommodate, when necessary, either two children or a mother nursing her own child. The general details and finishing of this block are similar to those in Block B.

Block D contains a laundry, with drying ground, completely fitted with Bradford's hand apparatus; a disinfecting chamber, fitted with Goddard and Massey's steam apparatus, an ambulance house, and a mortuary.

The drainage system is a dual one. The whole of the surface water, and so much of the rain water as could not on account of the fall of the ground be utilised, is discharged into a cesspool. Such of the rain water as it was possible to save is stored in an underground tank, from whence it is pumped for use in the laundry. The soil drainage is carried by sewers to an ejector station in the Fair Mile, whence it is discharged

ISOLATION HOSPITAL, HENLEY-ON-THAMES.

GROUND FLOOR PLAN

by Shone's ejectors into the Henley town system of sewerage.

The furniture, some of which has been specially designed by the architect, has been supplied by Messrs. Gregory and Co., of Regent Street. The contractors for the work were Messrs. Higgs and Hill, of London, and the architect, Mr. Keith D. Young.

## Figures and Tables

**Figure f1:**